# Effect of hypothermia on interleukin-1 receptor antagonist pharmacodynamics in inflammatory-sensitized hypoxic-ischemic encephalopathy of term newborns

**DOI:** 10.1186/s12974-018-1258-6

**Published:** 2018-07-30

**Authors:** Mathilde Chevin, Clémence Guiraut, Guillaume Sébire

**Affiliations:** Department of Pediatrics, McGill University, Research Institute of the McGill University Health Centre, 1001 Decarie Boulevard, (Glen site, Block E, M0.3211), Montreal, Quebec H4A 3J1 Canada

**Keywords:** Cerebral palsy, HT, IL-1Ra, Neonatal encephalopathy, Inflammation

## Abstract

**Background:**

Hypothermia is increasingly tested in several neurological conditions, such as neonatal encephalopathy, stroke, traumatic brain injury, subarachnoid hemorrhage, spinal cord injury, and neurological outcomes of cardiac arrest. Current studies aim to increase benefits of hypothermia with new add-on therapies including immunomodulatory agents. Hypothermia has been shown to affect the metabolism of commonly used drugs, including those acting on neuroimmune pathways.

**Objective:**

This study focuses on the effect of hypothermia on interleukin-1 receptor antagonist pharmacodynamics in a model of neonatal encephalopathy.

**Methods:**

The effect of hypothermia on (i) the tissue concentration of the interleukin-1 receptor antagonist, (ii) the interleukin-1 inflammatory cascade, and (iii) the neuroprotective potential of interleukin-1 receptor antagonist has been assessed on our rat model of neonatal encephalopathy resulting from inflammation induced by bacterial compound plus hypoxia-ischemia.

**Results:**

Hypothermia reduced the surface of core and penumbra lesions, as well as alleviated the brain weight loss induced by LPS+HI exposure. Hypothermia compared to normothermia significantly increased (range 50–65%) the concentration of the interleukin-1 receptor antagonist within the central nervous system. Despite this increase of intracerebral interleukin-1 receptor antagonist concentration, the intracerebral interleukin-1-induced tumor necrosis factor-alpha cascade was upregulated. In hypothermic condition, the known neuroprotective effect of interleukin-1 receptor antagonist was neutralized (50 mg/kg/12 h for 72 h) or even reversed (200 mg/kg/12 h for 72 h) as compared to normothermic condition.

**Conclusion:**

Hypothermia interferes with the pharmacodynamic parameters of the interleukin-1 receptor antagonist, through a bioaccumulation of the drug within the central nervous system and a paradoxical upregulation of the interleukin-1 pathway. These effects seem to be at the origin of the loss of efficiency or even toxicity of the interleukin-1 receptor antagonist when combined with hypothermia. Such bioaccumulation could happen similarly with the use of other drugs combined to hypothermia in a clinical context.

## Introduction

Pure hypoxia-ischemia (HI) and inflammatory-sensitized HI are the most prevalent clinical scenarios underlying neonatal encephalopathy (NE) of term newborns, one of the leading causes of neonatal death or cerebral palsy [1]. Neuroprotective treatments available against NE of term newborns consist in symptomatic cares and hypothermia (HT) [2, 3]. Ongoing researches focus on new add-on therapies in combination to HT to increase its neuroprotective effect [2, 4]. However, recent evidence demonstrated that HT can alter the pharmacokinetic and pharmacodynamic parameters of drugs and induces unexpected and sometimes adverse effects [5–8]. Our team and others recently showed that HT fails to counteract the IL-1 system [9, 10], which plays a key role in NE [11–14]. Interleukin-1 receptor antagonist (IL-1Ra) has already demonstrated a protective perinatal efficacy on several organs, especially the brain, exposed to inflammation induced by bacterial compounds and/or HI [11, 12, 15, 16]. These results support a potential neuroprotective benefit of IL-1Ra as a targeted add-on therapy to HT. An initial step in evaluating the effect of IL-1Ra in combination with HT is to test the effect of HT on its pharmacodynamics in this physiopathological context. Our hypothesis is that HT modifies the pharmacodynamic parameters of IL-1Ra under perinatal inflammatory and/or HI conditions. Our objectives will test the effect of HT on (i) the tissue concentration of IL-1Ra, including the central nervous system; (ii) the inflammatory cascade of the IL-1 system; and (iii) the neuroprotective potential of IL-1Ra.

## Material and methods

### Rat model

**Fig. 1:**
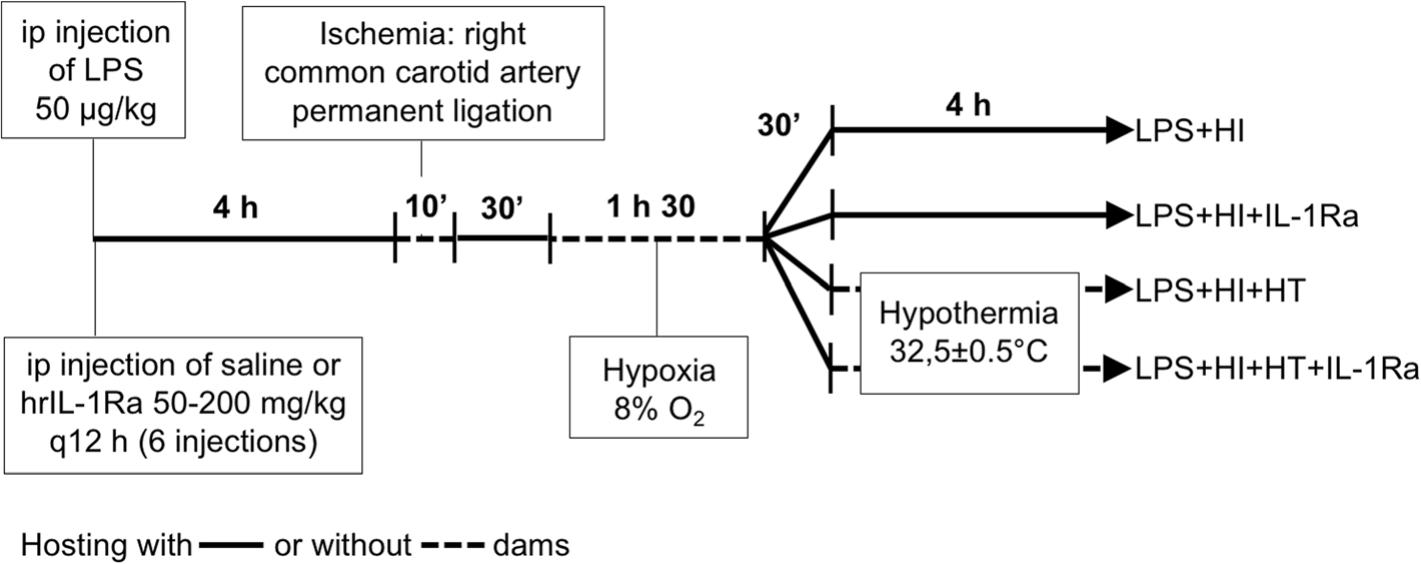
Experimental design. The first hrIL-1Ra (50–200 mg/kg) or saline injection was administrated 30 min before the ip injection of LPS from *Escherichia coli* (50 μg/kg) in pups at P12. Four hours later, the right common carotid artery was ligated, and hypoxia was induced (8% O_2_ for 1.5 h). Rat pups were subjected or not to hrIL-1Ra (50–200 mg/kg q12 h from P12 to P14) and treated or not by HT (32.5 °C ± 0.5 °C for 4 h). *Abbreviations*: HI, hypoxia-ischemia; HT, hypothermia; hrIL-1Ra, human recombinant of interleukin-1 receptor antagonist; ip, intraperitoneally; LPS, lipopolysaccharide from *Escherichia coli*; P, postnatal day

Our preclinical model was designed as previously described [9, 14, 15]. Briefly, pups at postnatal day (P) 5–7 were obtained from Charles River Laboratories (Saint-Constant, QC). At P12, they received a single intraperitoneal (ip) injection of lipopolysaccharide (LPS, 50 μg/kg diluted in 50 μl of pyrogen-free saline) from *Escherichia coli* (Sigma-Aldrich, ON). HI was induced 4 h after LPS administration by permanent ligation of the right common carotid artery followed by 8% O_2_ exposure at 36 °C for 1.5 h [9, 15, 17]. HT was induced 30 min after hypoxia, as previously described [9]. Briefly, pups were kept on a hot plate at 32 °C in order to lower their core body temperature until 32.5 °C ± 0.5 °C (Fig. 1). HT was maintained in a reproducible manner for 4 h. LPS+HI and LPS+HI+IL-1Ra pups stayed with the dam during the time their peers underwent HT [9].

Human recombinant (hr) IL-1Ra was used at a concentration of 50 or 200 mg/kg (diluted in 50 μl of pyrogen-free saline). Both doses are commonly used in the perinatal preclinical context to protect the organs against inflammation and/or HI [15, 16]. The first injection was given ip, 30 min before LPS injection. Five other injections were given every 12 h thereafter (Fig. 1). The end of hypoxia referred to as 0 h. Pups were euthanized at 4 h (which correspond to the end of HT), 24 h (P13), or 8 days (P20) post-HI. A total of 181 pups were included in the study. Pups were randomized in five experimental groups, namely 35 pups in LPS+HI condition, 32 pups in LPS+HI+HT condition, 24 pups in LPS+HI+IL-1Ra (50 mg/kg) condition, 52 pups in LPS+HI+HT+IL-1Ra (50 mg/kg), and 7 pups in LPS+HI+HT+IL-1Ra (200 mg/kg). Among all pups subjected to LPS+HI±HT±IL-1Ra (*n* = 181), the mortality rate was 17% (death occurred for all pups during hypoxia, except for 3 pups who died within 10 h following hypoxia). No significant difference was observed in the mortality rate between all experimental groups.

The experimental protocol was approved by the Institutional Animal Care Committee of the McGill University (protocol #2015-7691) in accordance with the Canadian Council on Animal Care guidelines: http://www.ccac.ca/en_/standards/guidelines.

### Cerebrospinal fluid (CSF) collection

CSF was collected by cisternal puncture of anesthetized rat pups at 4 or 24 h post-HI, as described [18, 19]. The mean volume of CSF collected was 28 μl (range 10–45 μl) with 96% of successful collection. CSF samples were kept frozen at − 80 °C. Immediately after CSF collection, rat pups were euthanized by decapitation, and their forebrain rapidly removed and frozen by immersion in methylbutane on dry ice.

### Histology

The brains were removed and fixed (paraformaldehyde 4%, glutaraldehyde 0.1%) at room temperature, paraffin-embedded, and cut in 5-μm slices using a microtome, as described [9, 15]. Hematoxylin-eosin (H&E) staining was performed to visualize brain injuries. Coronal sections were scanned, and the surface of the hemispheres were located at the epicenter of the infarct (Bregma from − 2.30 to − 2.50 mm), as previously described [9, 14, 15]. Core versus penumbra areas of brain infarcts were defined as previously described [9, 15]. Briefly, core injuries were associated with infarcted areas bearing cavitary lesions, whereas penumbra injuries were identified as regions surrounding the core where pycnotic neurons and/or loss of normal neuronal architecture were observed [9, 15].

### ELISA

Protein extracts were prepared from right hemisphere forebrains as previously described [9, 14, 15]. ELISAs were performed on these protein extracts using ELISA Kits (R&D System, MN, USA), as previously described [9, 14, 15].

### Behavioral test

The open field test was used to determine spontaneous locomotor activity and exploratory behavior of juvenile rats (P20), as described previously [20]. The following parameters were assessed in the open field apparatus using Any-Maze Video Tracking System™ (IL, USA) software: total distance traveled during the test period, mobile time, time in the center, and number of square visited.

### Data analysis

Statistical analyses were performed using IBM Statistics 24 (SPSS) and GraphPad software version 6.02. The data are presented as the mean ± standard error of the mean (SEM). Normality was assessed across experimental conditions. Data were analyzed by independent samples *t* test or one-way analysis of variances (ANOVA) with Tukey’s HSD test. Mann-Whitney *U* test was used when data were not normally distributed. Male and female data were combined, because no significant interaction was observed between sex and treatment. The statistical significance level was set at *p* ≤ 0.05.

## Results

### Effect of HT on hrIL-1Ra titers within the tissues of interest

**Fig. 2:**
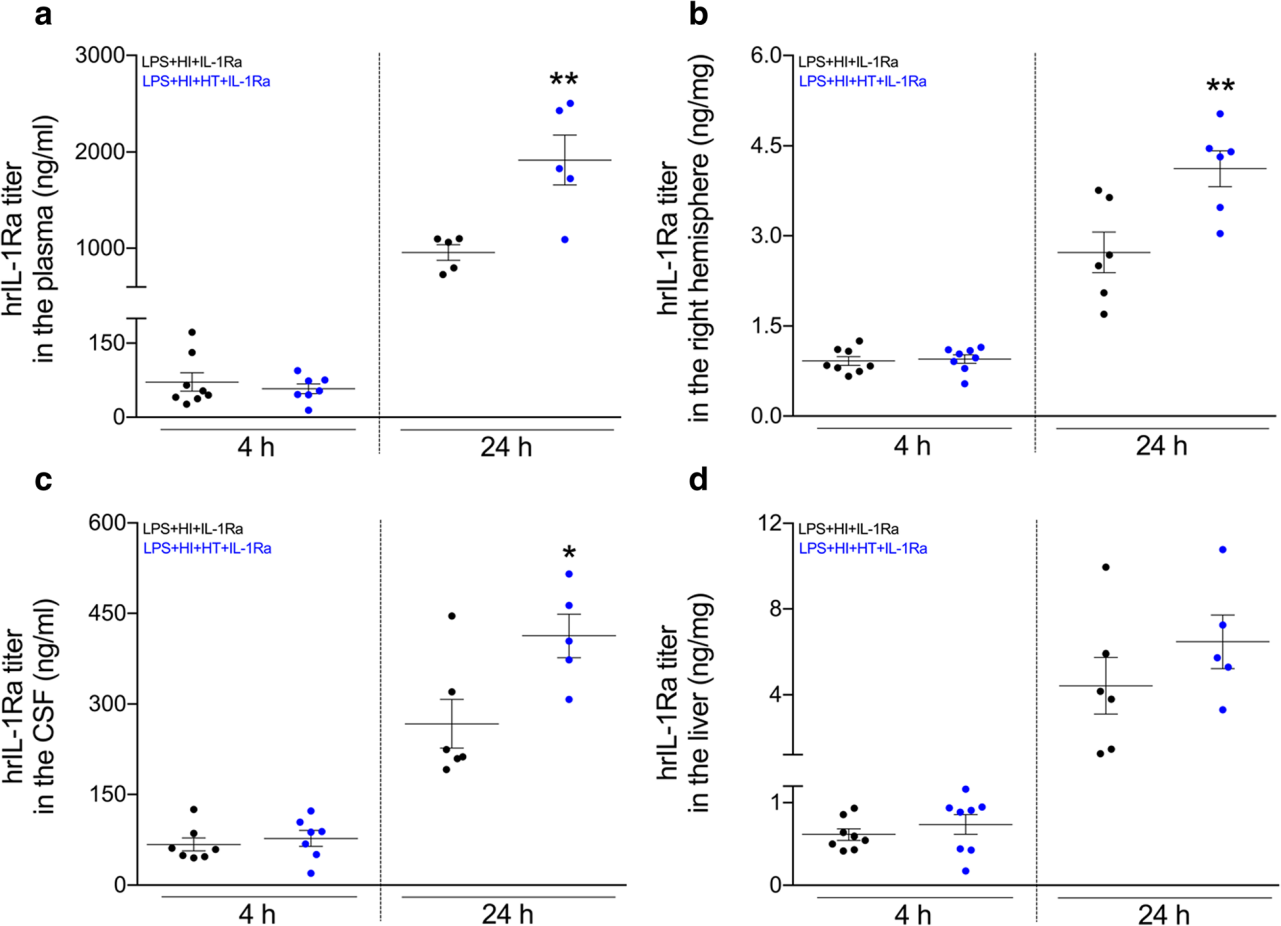
hrIL-1Ra titers within tissues of interest from pups exposed to LPS+HI+IL-1Ra±HT. hrIL-1Ra titers measured by ELISA at 24 h post-HI were increased within the plasma (**a**), right cerebral hemisphere (**b**), and CSF (**c**) in LPS+HI+HT+IL-1Ra (50 mg/kg) as compared to LPS+HI+IL-1Ra (50 mg/kg) condition. The concentrations of hrIL-1Ra were similar in both conditions at 4 h post-HI, as well as at 24 h post-HI within the liver (**d**). The number (*n*) of rats used was LPS+HI+IL-1Ra (*n* = 5–8 from 4 litters) and LPS+HI+HT+IL-1Ra (*n* = 5–8 from 4 litters). The bars indicate the mean ± SEM. *p ≤ 0.05, **p ≤ 0.01; independent *T* test. *Abbreviations*: CSF, cerebrospinal fluid; HI, hypoxia-ischemia; HT, hypothermia; hrIL-1Ra, human recombinant of interleukin-1 receptor antagonist; LPS, lipopolysaccharide from *Escherichia coli*

At 4 h post-HI, HT did not modify the titer of hrIL-1Ra, at the dose of 50 mg/kg, within the organ tested, namely plasma, liver, CSF, and right forebrain hemisphere exposed to LPS+HI (Fig. 2). At 24 h post-HI, HT induced a significant increase (50 to 65%) of the hrIL-1Ra titers within the plasma, CSF, and right forebrain hemisphere exposed to LPS+HI (Fig. 2a–c).

### Effect of HT+hrIL-1Ra (50 mg/kg) on the inflammatory cascade-induced by LPS+HI exposure

**Fig. 3:**
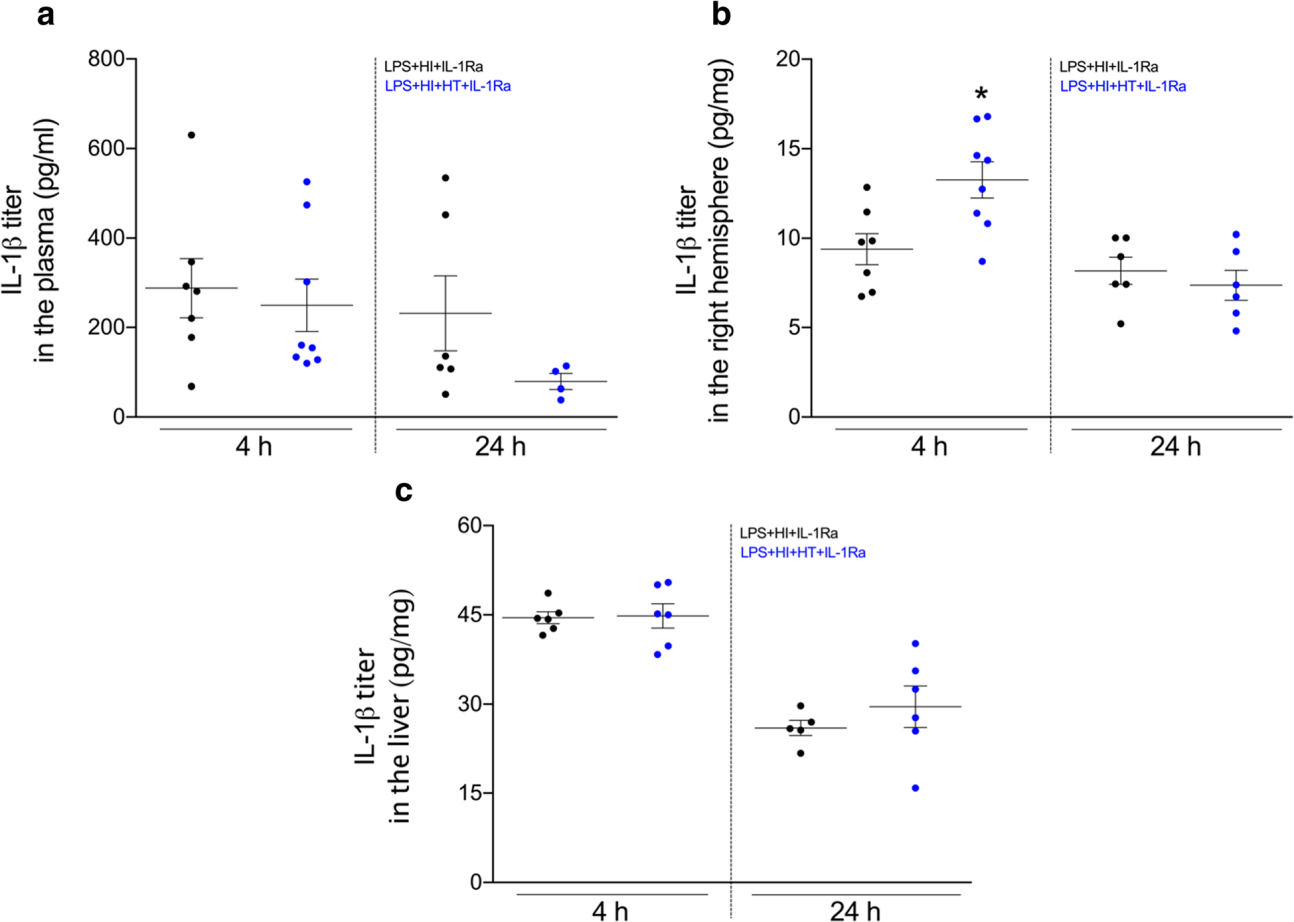
IL-1β expression within tissues of interest from pups exposed to LPS+HI+IL-1Ra±HT. IL-1β concentration measured by ELISA at 4 h and 24 h post-HI within the plasma (**a**), right cerebral hemisphere (**b**), and liver (**c**) in LPS+HI+IL-1Ra (50 mg/kg) and LPS+HI+HT+IL-1Ra (50 mg/kg) conditions. HT increased the expression of IL-1β within the right hemisphere at 4 h post-HI (**b**). The number (*n*) of rats used was LPS+HI+IL-1Ra (*n* = 5–7 from 4 litters) and LPS+HI+HT+IL-1Ra (*n* = 4–8 from 4 litters). The bars indicate the mean ± SEM. *p ≤ 0.05; independent *T* test. *Abbreviations*: HI, hypoxia-ischemia; HT, hypothermia; hrIL-1Ra, human recombinant of interleukin-1 receptor antagonist; IL-1β, interleukin-1β; LPS, lipopolysaccharide from *Escherichia coli*

**Fig. 4:**
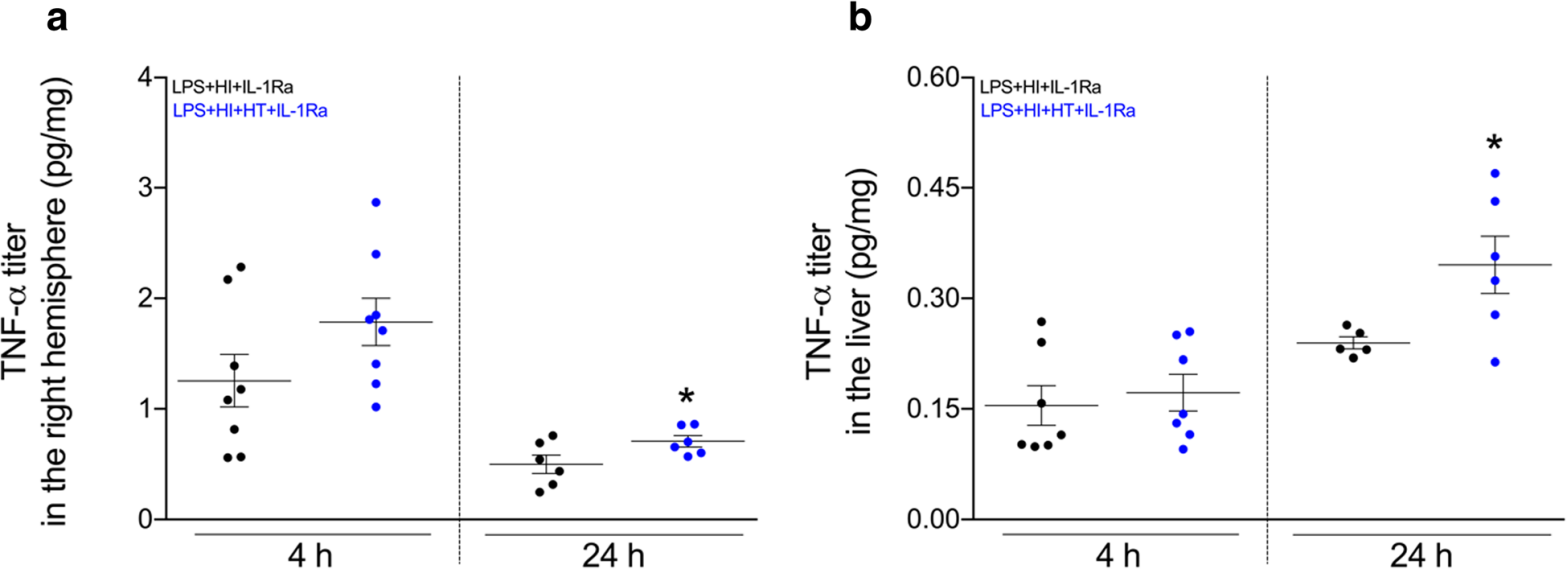
TNF-α titers within tissues of interest from pups exposed to LPS+HI+IL-1Ra±HT. TNF-α concentrations measured by ELISA were increased at 24 h post-HI within the right cerebral hemisphere (**a**) and the liver (**b**) in LPS+HI+HT+IL-1Ra (50 mg/kg) as compared to LPS+HI+IL-1Ra (50 mg/kg) conditions. The TNF-α titers were similar in both conditions at 4 h post-HI. The number (*n*) of rats used was LPS+HI+IL-1Ra (*n* = 5–8 from 4 litters) and LPS+HI+HT+IL-1Ra (*n* = 6–8 from 4 litters). The bars indicate the mean ± SEM. *p ≤ 0.05; independent *T* test. *Abbreviations*: HI, hypoxia-ischemia; HT, hypothermia; hrIL-1Ra, human recombinant of interleukin-1 receptor antagonist; LPS, lipopolysaccharide from *Escherichia coli*; TNF-α, tumor necrosis-α

IL-1Ra administration interferes with the autocrine loop of IL-1β synthesis and shuts down the downstream inflammatory cascades including TNF-α production [11, 15, 21, 22]. In HT conditions at 4 and 24 h post-HI, hrIL-1Ra (50 mg/kg) failed to counteract these pathways (Fig. 3), or conversely induced paradoxical upregulations of the IL-1β production at 4 h post-HI (Fig. 3b), and of the TNF-α production at 24 h post-HI in the LPS+HI-exposed right hemisphere (Fig. 4a).

### Dose-dependent neurotoxic effect of hrIL-1Ra added to HT

**Fig. 5:**
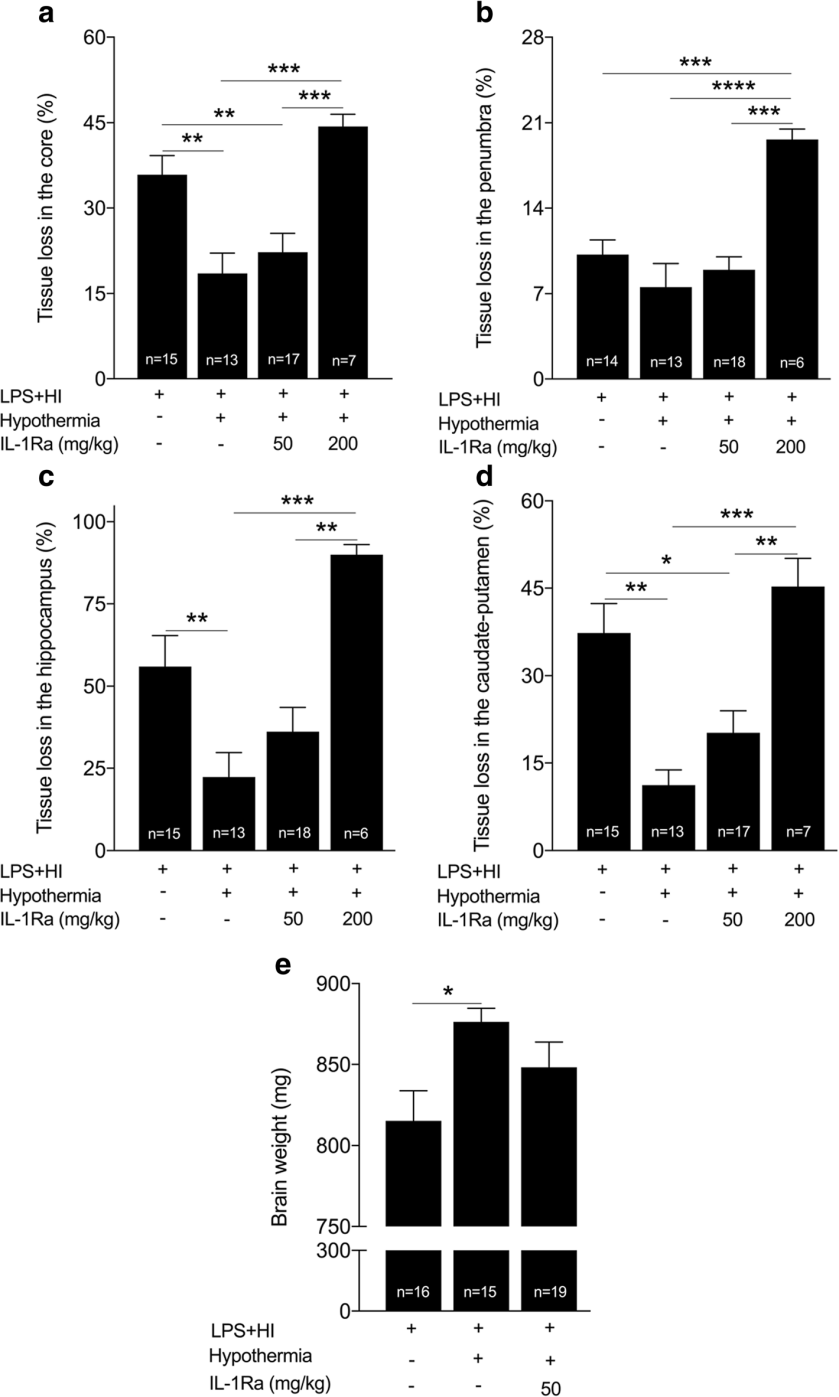
Comparison of the extent of brain injuries between LPS+HI±HT±IL-1Ra conditions. Comparisons of the extent of core and penumbra injuries (within the neocortex, hippocampus, and caudate-putamen) between pups exposed to LPS+HI±HT±IL-1Ra (50–200 mg/kg) by H&E staining of the right forebrains at P20. HT reduced the surface of core and penumbra lesions (**a**–**d**), as well as alleviated the brain weight loss observed after LPS+HI exposure (**e**). The surface of core and penumbral lesions were similar in LPS+HI+HT+IL-1Ra (50 mg/kg) as compared to LPS+HI+HT condition (**a**–**d**). HT+hrIL-1Ra (200 mg/kg) increased the extent of penumbra injury as compared to the LPS+HI condition (**b**), as well as core and penumbral injuries as compared to LPS+HI+HT and LPS+HI+HT+IL-1Ra (50 mg/kg) (**a**–**d**). The number (*n*) of rats used was LPS+HI (*n* = 14–16 from 9 litters), LPS+HI+HT (*n* = 13–15 from 9 litters), LPS+HI+HT+IL-1Ra 50 mg/kg (*n* = 17–19 from 9 litters), and LPS+HI+HT+IL-1Ra 200 mg/kg (*n* = 6–7 from 3 litters). The bars indicate the mean ± SEM. *p ≤ 0.05, **p ≤ 0.01, ***p ≤ 0.001, ****p ≤ 0.0001; one-way ANOVA. *Abbreviations*: HI, hypoxia-ischemia; HT, hypothermia; hrIL-1Ra, human recombinant of interleukin-1 receptor antagonist; LPS, lipopolysaccharide from *Escherichia coli*

**Fig. 6:**
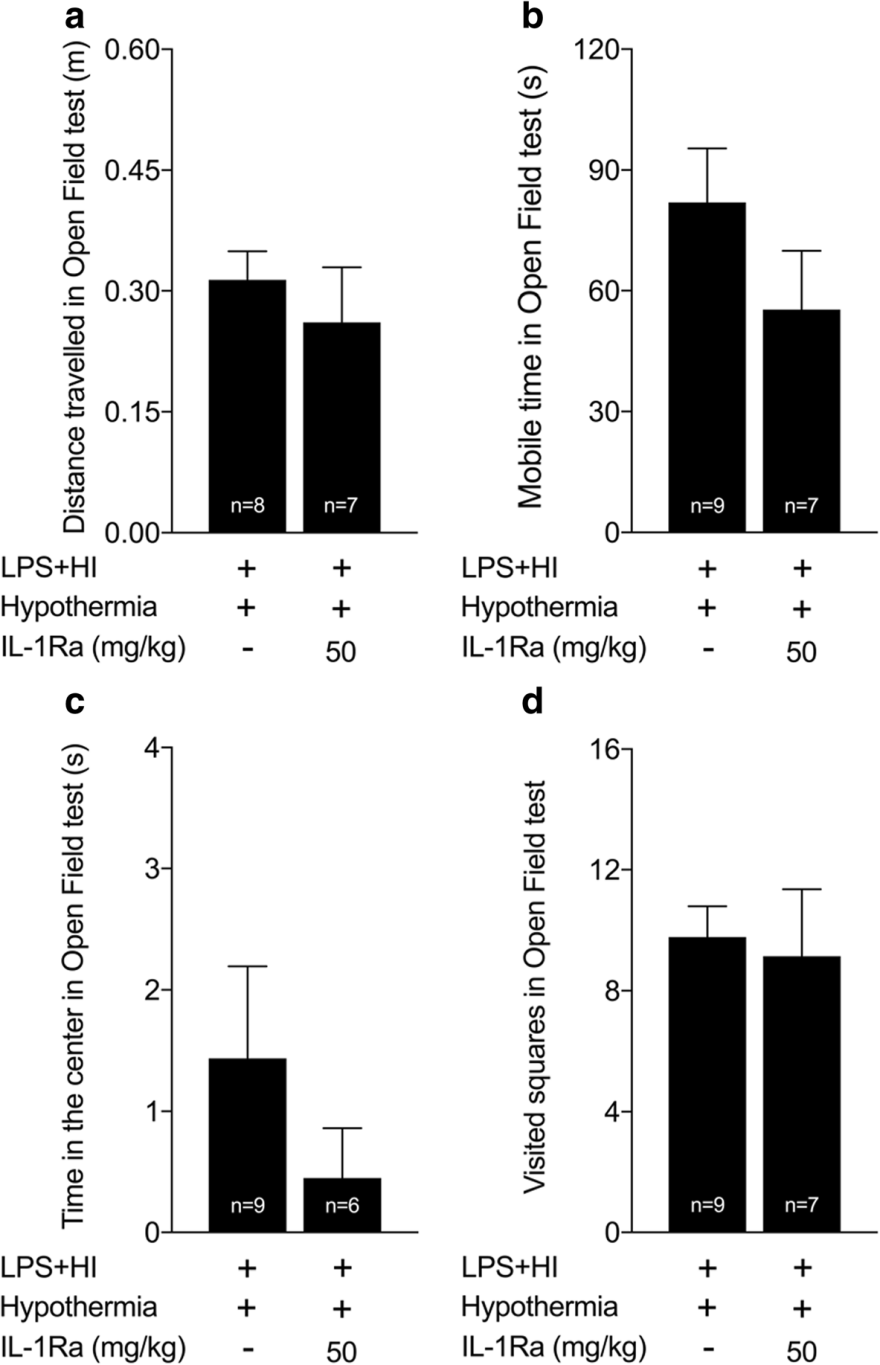
Open field experiment at P20 in pups exposed to LPS+HI+HT±IL-1Ra (50 mg/kg). No difference was observed between the two conditions for the different open field parameters tested: the distance traveled (**a**), the mobile time (**b**), the time in the center (**c**), and the visited squares in the apparatus (**d**). The number (*n*) of rats used was LPS+HI+HT (*n* = 8–9 from 6 litters) and LPS+HI+HT+IL-1Ra 50 mg/kg (*n* = 6–7 from 5 litters). Independent *T* test. *Abbreviations*: HI, hypoxia-ischemia; HT, hypothermia; hrIL-1Ra, human recombinant of interleukin-1 receptor antagonist; LPS, lipopolysaccharide from *Escherichia coli*

HT alone exerted a neuroprotective effect on the extent of LPS+HI-induced core (Fig. 5a) and penumbral injuries (Fig. 5b–d). HT also protected against the loss of brain weight observed in such condition (Fig. 5e). hrIL-1Ra at the dose of 50 mg/kg did not provide any neuroprotective added value when combined to HT (Fig. 5a–d). hrIL-1Ra at the dose of 200 mg/kg increased LPS+HI-induced penumbral—but not core—injuries (Fig. 5b). Open field experiments in juvenile rats (P20) did not show any difference between LPS+HI+HT versus LPS+HI+HT+hrIL-1Ra (50 mg/kg) conditions (Fig. 6a–d).

## Discussion

Our results showed that HT altered the pharmacodynamic parameters of hrIL-1Ra in our model of NE-induced by inflammation plus HI. HT increased the concentration of hrIL-1Ra (at 24 h post-HI) within the LPS+HI-exposed plasma, CSF, and forebrain. Paradoxically, this effect was not associated with an IL-1Ra-induced anti-inflammatory effect on the IL-1 system. We also observed a lack of effectiveness of the combination of hrIL-1Ra with HT, as compared to sole hrIL-1Ra in the same model of LPS+HI-induced NE [14, 15].

According to the pharmacokinetic study performed in a rat model of arthritis [23], and also taking into account the short half-life (4–6 h) of IL-1Ra, it is unlikely that an accumulation of IL-1Ra would be due in our experimental design to the repeated administration of IL-1Ra every 12 h. We hypothesize that the blood brain barrier (BBB) dysfunction induced by LPS+HI exposures might increase over time, with a more important BBB leak at 24 h (allowing the IL-1Ra to diffuse within the brain) as compared to 4 h post-HI. Few studies dealt with the impact of HT on the pharmacokinetic and pharmacodynamic of drugs used in the human neonatal context. However, it was shown that several drugs—e.g., isoflurane, morphine, ligands of β1 and β2 adrenoreceptors—had reduced metabolism and clearance on HT as compared to non-HT condition [5, 6]. Affinity between ligands and their cognate receptors as well as alterations of downstream signaling are also reported on HT [5, 6, 8]. Our results suggest that the bioaccumulation of hrIL-1Ra within the brain and CSF in LPS+HI+HT condition might result from a decreased clearance of hrIL-1Ra and/or from a decreased affinity of hrIL-1Ra for the IL-1R, and also possibly from the blockade of the IL-1R signaling pathway. hrIL-1Ra is rapidly eliminated (half-life of 4–6 h) mainly by the kidney through glomerular filtration (GFR) [24]. It is known in human studies that the GFR is decreased under hypothermic condition [5, 6]. Besides, acute kidney injury can be associated to HI encephalopathy in the term neonate [25, 26]. Hence, HI could potentially affect the renal filtration, especially in the HT condition, and decrease the clearance of IL-1Ra.

The increased hrIL-1Ra bioaccumulation in HT condition might explain the switch from protective [14, 15] to toxic effects of our highest dose of hrIL-1Ra (200 mg/kg/12 h for 72 h). hrIL-1Ra (200 mg/kg/12 h for 72 h) might reach in HT condition a toxic concentration within the brain inducing non-specific ligand-receptors interactions deleterious for neural cells.

This study has some limitations. The concentration of hrIL-1Ra was assessed only at 4 and 24 h post-HI. In future experiments, blood samples could be taken at additional time-points to study in more detail the pharmacology of this drug. However, to our knowledge, this is the first study focusing on the pharmacology of IL-1Ra in neonatal rats.

## Conclusion

Our study addresses for the first time the impact of HT on hrIL-1Ra pharmacodynamics. HT might decrease the clearance of hIL-1Ra, inducing its bioaccumulation and loss of efficiency within the brain [11, 14, 15, 22, 27]. According to this hypothesis, current and future studies aiming to develop HT therapies—as already performed in neurological conditions, such as neonatal encephalopathy, stroke, traumatic brain injury, subarachnoid hemorrhage, spinal cord injury, and neurological outcomes of cardiac arrest [28–30]—should take into account the pharmacokinetic and pharmacodynamic impact of HT and the inherent modification of the safety profile of drugs.

## Abbreviations

TNF-α: Tumor necrosis factor α
CSF: Cerebrospinal fluid
GFR: Glomerular filtration
HI: Hypoxia-ischemia
hrIL-1Ra: Human recombinant of interleukin-1 receptor antagonist
HT: Hypothermia
IL: Interleukin
ip: Intraperitoneally
LPS: Lipopolysaccharide from *Escherichia coli*
NE: Neonatal encephalopathy
P: Postnatal day
SEM: Standard error of the mean
